# The Medical Profession and the War Office

**Published:** 1915-09-11

**Authors:** 


### THE MEDICAL PROFESSION AND THE WAR OFFICE.

These are times when the critic is indeed abroad in the land, and whatever other institutions have escaped his edge the War Office has certainly not lacked for censors. The medical profession has necessarily been brought into close association with the official organisation, and it would be idle to deny that the experience has not generated some measure of friction. We must allow, further, that our own columns have indicated certain points of difference, and we trust that our comments have had a foundation of facts, and have been temperately expressed. The aim of all good citizens just now is to help, not to hinder, those charged with authority. One way of doing this is to present points of view which have not been appreciated by the official mind; another is to recognise the good will and courtesy with which suggestions have been received. This latter might perhaps be practised more frequently, and we propose to offer here a small contribution in this direction.

First, it is well to record the action of the War Office in relation to the various War Emergency Committees which have been organised by the profession with a view to secure a sufficiency of medical officers for the Army. These committees certainly contain a number of distinguished men, but official machinery is not always tolerant of distinction outside the limits of its own operations. Nor does such distinction always imply the habit of co-operative enterprise and working. Hence, as experience daily teaches, the professional and the amateur often fail to accommodate successfully their individual peculiarities. In the present instance it is satisfactory to know that there has been no friction of this order. The military authorities have met the representatives of the profession quite frankly and fairly. So far from the reticence which the official world is apt to practise, there has been an unqualified revelation —given under the seal of confidence—of the position at the moment, and also of the anticipated needs of the future. Again, the authorities, so far from desiring to interfere in the schemes and operations of the committees, have left them a free hand to deal with the situation, and have accorded to them a complete and official recognition. That this course has been dictated by wisdom we do not doubt, but at the same time it is well to remember that here at least there has been no want of consideration and courtesy for the representatives of the medical profession. These have been recognised as helpers, and the help has been appreciated.

It has to be remembered also that the services rendered by medical men engaged in active service have not failed to secure attention. Both by formal announcement in dispatches and by individual distinctions the Army doctor has been proclaimed an essential and valuable part of the fighting organisation, and the triumphs of the sanitary service are told by all men. To some all this may seem natural and inevitable, but others with longer memories will appreciate the present all the more that they are able to recall a very different past. That there are points less open to congratulation we do not deny, and on some of these it may be necessary still to insist. But our immediate purpose is to recall to our own mind and to those of our readers experiences which go to show that the medical profession has conquered for itself a great position in this time of national crisis, and that of this position the representatives of the War Office are by no means unmindful.

